# A case with type Ⅱ vesicouterine fistula

**DOI:** 10.1002/ccr3.5284

**Published:** 2022-02-20

**Authors:** Zhi‐peng Yan, Cheng‐cheng Wang, Yang‐yang Wang, Sheng‐Tian Zhao

**Affiliations:** ^1^ Shandong Provincial Hospital Affiliated to Shandong First Medical University Jinan China

**Keywords:** cesarean section, cystoscopy, hematuria, laparoscopy, vesicouterine fistula

## Abstract

Here, we report a 41‐year‐old female with type II VUF after hysteromyomectomy.We report the diagnosis of VUF by imaging method, and provide a feasible treatment for this complication after pelvic surgery.

## Graphical abstract

As a urogenital fistula, vesicouterine fistula (VUF) is more common in female patients after pelvic surgery. In this study, we report a 41‐year‐old woman with type II VUF after hysteromyomectomy.

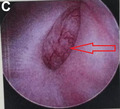

## 1 INTRODUCTION

With the large‐scale development of cesarean section, VUF has an upward trend. Jówik^1^ divides VUF into three types according to symptoms: Type I shows periodic hematuria, no vaginal bleeding, and completely controllable urine (without vaginal leakage); type II shows that urine and menstrual blood flow in both directions through fistula, and vaginal leakage and periodic hematuria can be seen; and type III is characterized by normal menstruation with or without vaginal leakage. This case belongs to type II VUF. We report the diagnosis of VUF by imaging method and provide a feasible treatment for this complication after pelvic surgery.

## 2 CASE HISTORY/EXAMINATION

**FIGURE 1:**
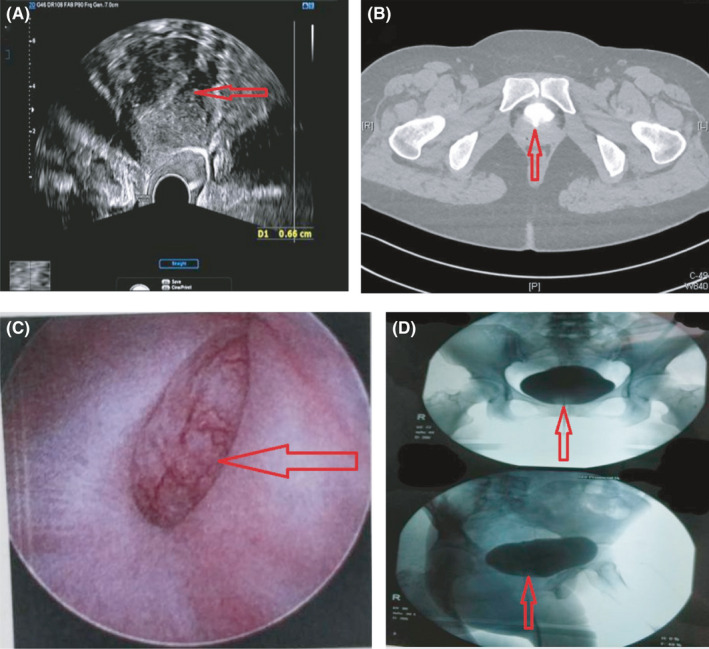
(A) Ultrasound showed that the continuity of the anterior wall of the cervix was interrupted at the level of the internal cervix, the defect was 0.55 cm wide, and the cervical canal communicated with the bladder. (B) Enhanced urography showed speckled high‐density lesions in the uterus below the bladder. (C) Cystoscopy showed that bladder wall defect was visible above the triangle of bladder, with a range of about 3 cm × 2 cm. D Methylene blue +contrast medium bladder instillation test showed slight vaginal leakage

A 41‐year‐old female patient came to the hospital for treatment because of “hematuria during menstruation with vaginal leakage for 2 years.” The patient underwent a “hysteromyomectomy” two years ago. After the operation, it was found that there was obvious abdominal pain and gross hematuria during menstruation and occasional vaginal leakage. She tried to relieve her symptoms by placing an IUD, but the symptoms of abdominal pain and periodic hematuria did not improve. Routine urine examination in our hospital showed the following: red blood cells (++++); ultrasound showed that the continuity of the anterior wall of the cervix was interrupted at the level of the internal cervix, the defect was 0.55 cm wide, and the cervical canal communicated with the bladder (Figure 1A); enhanced urography showed speckled high‐density lesions in the uterus below the bladder (Figure 1B). Cystoscopy showed that bladder wall defect was visible above the triangle of bladder, with a range of about 3 cm × 2 cm (Figure 1C); methylene blue +contrast medium bladder instillation test showed slight vaginal leakage (Figure 1D). Considering that fistula exists for 2 years, conservative treatment cannot cure it. Laparoscopic repair of bladder and uterine fistula was performed. During the operation, the VUF was about 3 cm, and the scar tissue around the fistula was removed. Pathological examination at the cutting edge showed collagen fiber tissue, and a small amount of endometrial tissue was locally seen. Postoperative patients were given indwelling catheter and antibiotics and gonadotropin drugs. Follow‐up for 5 months showed no vaginal leakage and periodic hematuria.

## 3 DISCUSSION

VUF accounts for only 1%–4% of all urogenital fistulas, and the increase in cesarean section is the main reason.^2^, ^3^ According to BHAT TACHARJEE,^4^ the rate of VUF caused by cesarean section is as high as 83%~93%. Tancer et al.^5^ reported that only 92 cases of VUF were reported worldwide from 1908 to 1986, and 64 new cases were reported from 1986 to 1997^6^; with the development of cesarean section and the improvement of nursing technology, the relative frequency of urogenital fistula has a changing trend. The most common cause of VUF is cesarean section, especially repeated cesarean section.^7^ VUF may cause infection, bacteremia, infertility, abortion, and other complications. It is even reported that long‐term VUF makes fetus spontaneously flow from uterus to bladder to form stillbirth.^8^ In this case, it is confirmed that the fistula is located near the cervix, where the sphincter function is good and persistent vaginal leakage will not occur; the pressure in uterine cavity is greater than that in bladder, which is the cause of periodic hematuria caused by menstrual blood entering bladder during menstruation.^9^ The diagnosis of VUF was mainly based on methylene blue staining test, cystoscopy, cystography, ultrasound (US), CT, and MRI.^10^ Cystoscopy and hysterography are considered by some scholars as the gold standard for the diagnosis of VUF. In this case, we used cystoscopy and cystography to determine that the fistula exists between the upper part of the bladder triangle and the anterior wall of uterus near the cervix. SMAYRA et al.^11^ held that enhanced CT is a good method to show fistula for low VUF, and when the existence of high fistula is suspected, spiral CT plain scan with three‐dimensional reconstruction function can be performed after hysterography. Cystoscopy is important for fistula location and its location in the bladder triangle, but it does not determine the upper pole of the fistula. Other diagnostic methods have limitations. Through literature search, it is found that MRI can achieve 100% accuracy in diagnosis. Compared with enhanced CT, MRI also has the advantages of noninvasive and nonionizing radiation, and MRI is regarded as the gold standard for diagnosis and treatment planning.^12^, ^13^ Therefore, it is recommended that MRI should be used to determine VUF first, and then, cystoscopy +contrast intravesical instillation test should be used to locate fistula. At present, there is no uniform treatment standard for VUF, and the treatment plan includes conservative treatment and surgical repair. When fistula is found after delivery, it is recommended to treat it conservatively through bladder intubation for at least 4–8 weeks and use oral contraceptives or other gonadotropin drugs to induce amenorrhea to promote fistula healing.^14^ ROUZI^15^ reported a case of successful cure of fistula after 6 weeks of Foley catheter catheterization. He believes that even if the fistula does not heal spontaneously, it will not affect the effect of surgical intervention. Laparoscopic repair has been proposed as an effective choice for repairing VUF. After repair, omentum or properitoneal fat is used as septal tissue to separate uterus from bladder to prevent recurrence of fistula^16^; compared with open surgery, laparoscopic fistula repair has the advantages of less trauma, quick recovery, and beautiful wound, which can be regarded as the standard VUF surgery. In recent years, the development of robot technology has also reported related operations.^17^ No matter what surgical method is used, the basic surgical principle of repairing VUF should be extensive resection of scar tissue around fistula, suturing of the fistula with absorbable sutures in multiple layers, elimination of dead space as much as possible, and prevention of hematoma formation. For patients with large fistula (>2 cm) and obvious adhesion of local scar tissue, indwelling catheter and cystostomy tube at the same time after operation are more conducive to the healing of fistula and bladder.^18^

## 4 CONCLUSIONS

In this study, we present a clinical case report, type Ⅱ VUF, and it is recommended that MRI examination +cystoscopy + contrast medium bladder perfusion test be used for qualitative and localized diagnosis before operation. The treatment plan for urogenital fistula is to take active intervention measures. When fistula is found after delivery or operation, it is recommended to carry out conservative treatment for at least 4–8 weeks through bladder intubation and promote fistula healing through oral gonadotropin drugs. If fistula is too large (>2 cm), surgical repair should be carried out as soon as possible to prevent the formation of old fistula.

## CONFLICT OF INTEREST

There are no conflicts of interest in the paper.

## AUTHOR CONTRIBUTION

ZP‐Y contributed to research conception and drafted the manuscript. CC‐W contributed to literature search and review. YY‐W participated in data collection. ZP‐Y and CC‐W should be considered joint first authors. All authors have approved the submitted manuscript and accepted all revisions.

## CONSENT

Written informed consent has been obtained from the patient.

## Data Availability

The data that support the findings of this study are available in PubMed. These data were derived from the following resources available in the public domain: https://pubmed.ncbi.nlm.nih.gov
